# Supplementary figures and images for: Toxoplasma infection in male mice alters dopamine-sensitive behaviors and host gene expression patterns associated with neuropsychiatric disease
Source: PLoS Negl Trop Dis. 2022 Jul 20;16(7):e0010600. doi: 10.1371/journal.pntd.0010600 (PMC9342775; doi:10.1371/journal.pntd.0010600)

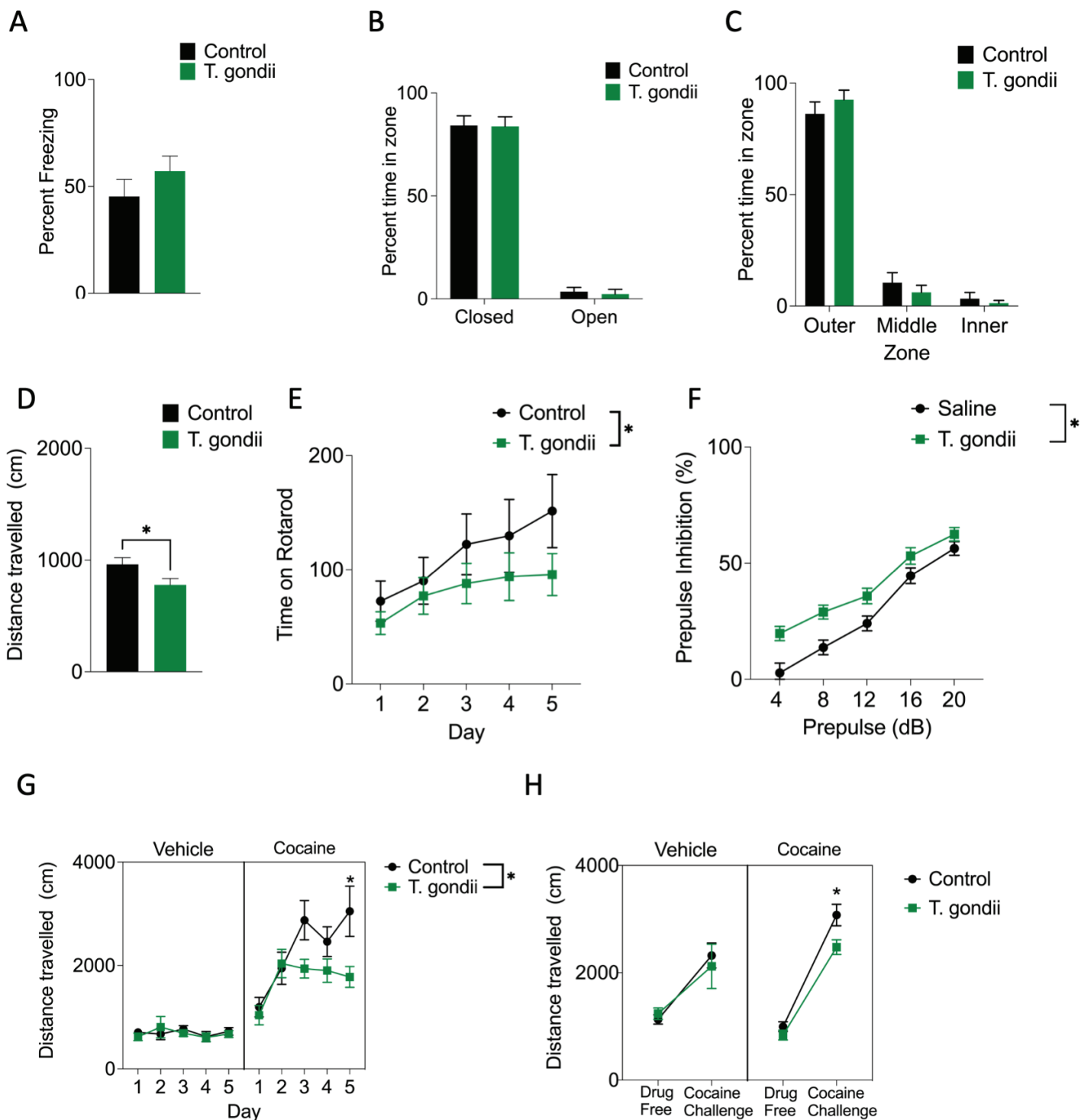

Supplement: S1 Fig — A—Infected mice are not impaired in a contextual fear conditioning (t-test, p = 0.28) B-C- Infected mice did not show altered anxiety behaviors in the elevated plus maze (Two-way ANOVA No significant main effect of group (p = 0.83) or region by group interaction (p = 0.92) (B) or open field test (Two way ANOVA No significant main effect of group (p = 0.99) or group by zone interaction (p = 0.34)) (C). D- infected mice exhibited a significant reduction in distance travelled in the open field (t-test t(18) = 2.24, p = 0.038). E–There was also a significant decrease in the time spent on the rotarod compared to control mice (Repeated measures ANOVA, main effect of group F(4,90) = 5.1, p = 0.026. group by session interaction p = 0.89. F–A reduction in prepulse inhibition was observed in infected mice compared to controls (Repeated measures ANOVA main effect of group F(4,90) = 31.69, p ≤ 0.0001 group by intensity interaction p = 0.45). G-H–As was observed with the CEP strain, infected mice had a blunted locomotor response to repeated cocaine administration significant group by drug treatment effect F(1,80) = 8.09, p = 0.0057)(G) however, there was no significant group or group type interactions (p’s ≥ 0.14) during a subsequent cocaine challenge one week following sensitization (H). (PDF) [file pntd.0010600.s001.pdf]

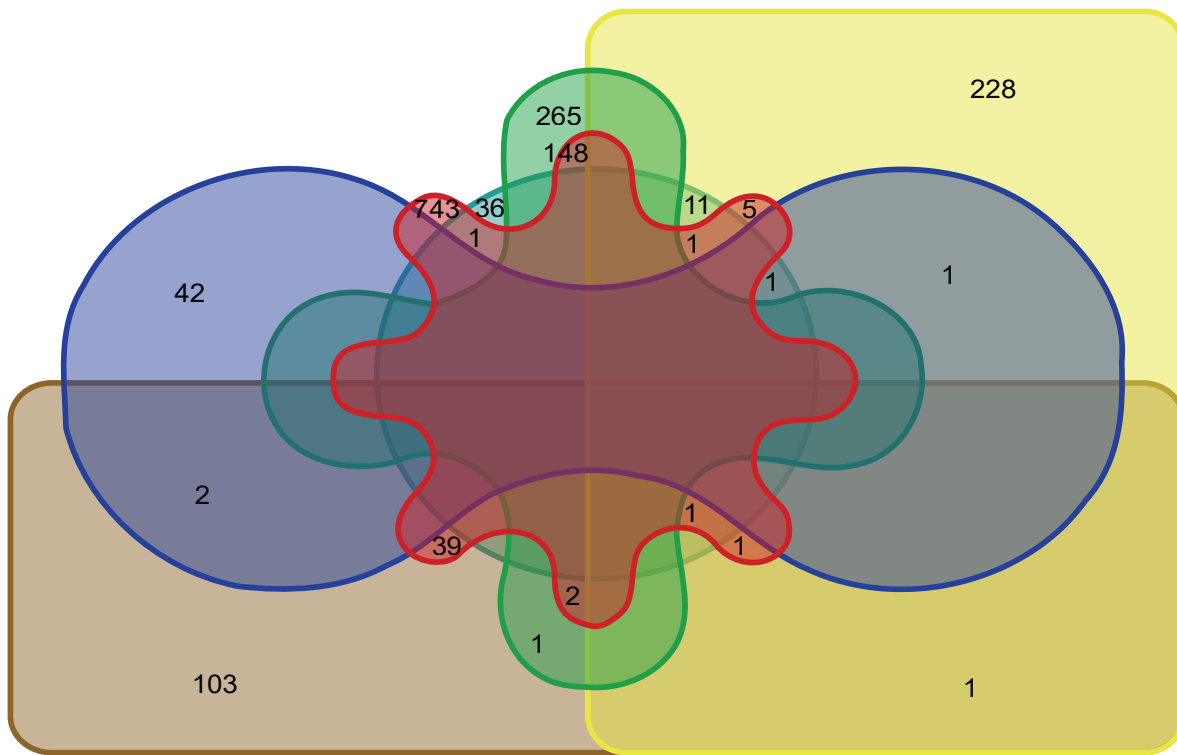

Search Key

- Neuro
- Behavior
- Synap
- Memory
- Locomot
- Brain

Supplement: S2 Fig — (PDF) [file pntd.0010600.s002.pdf]

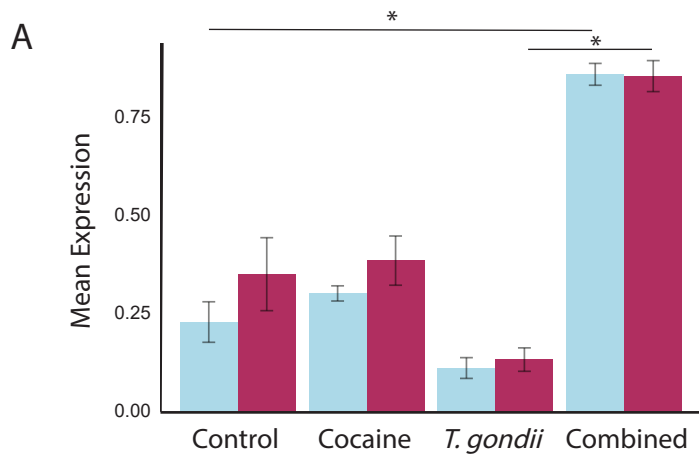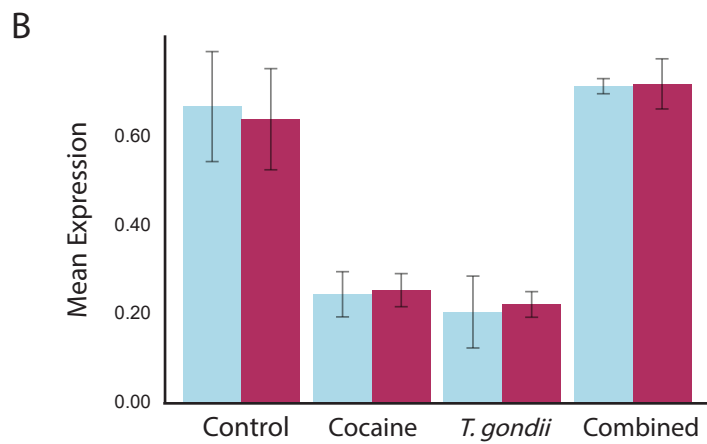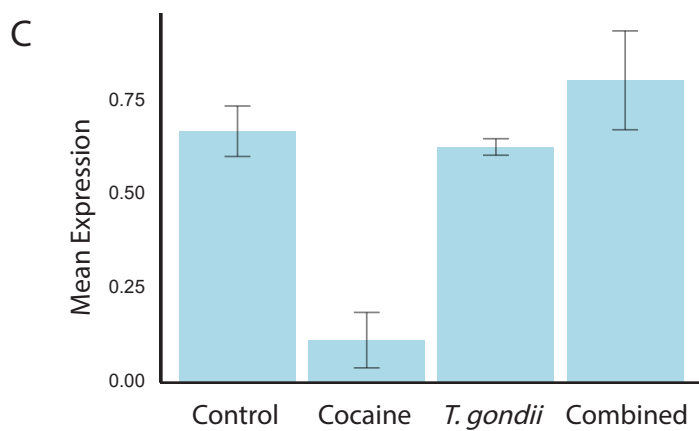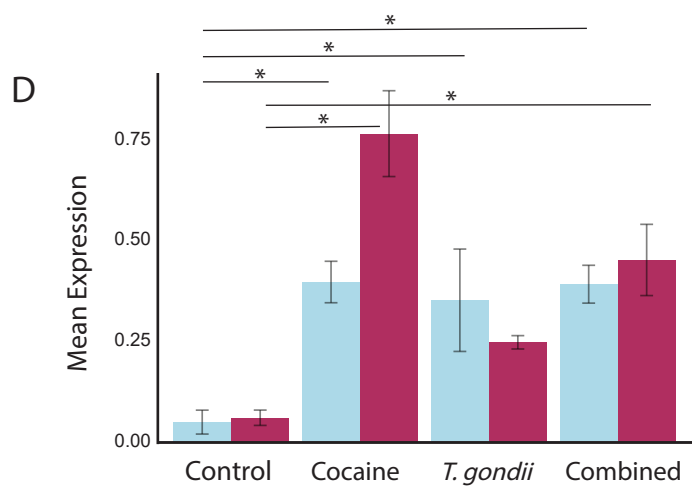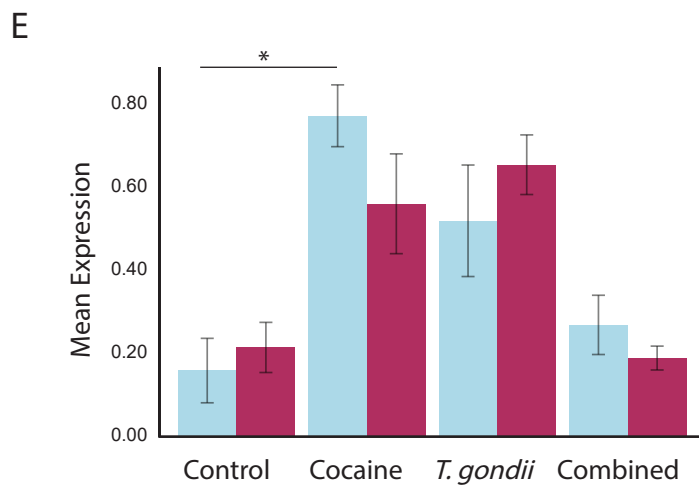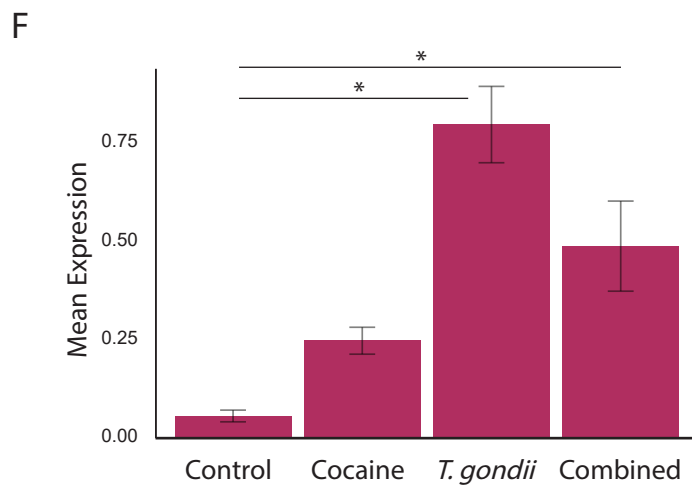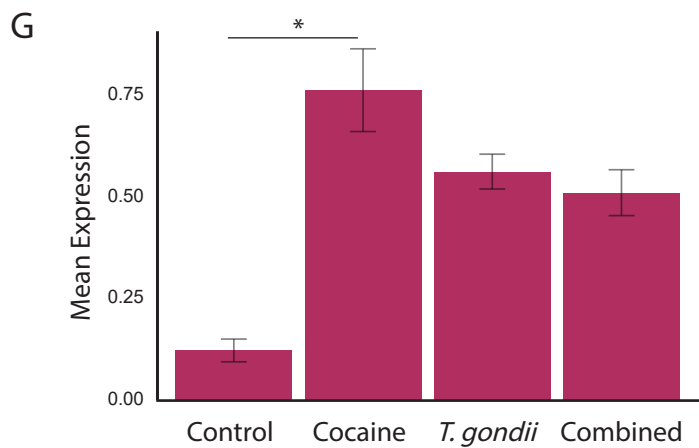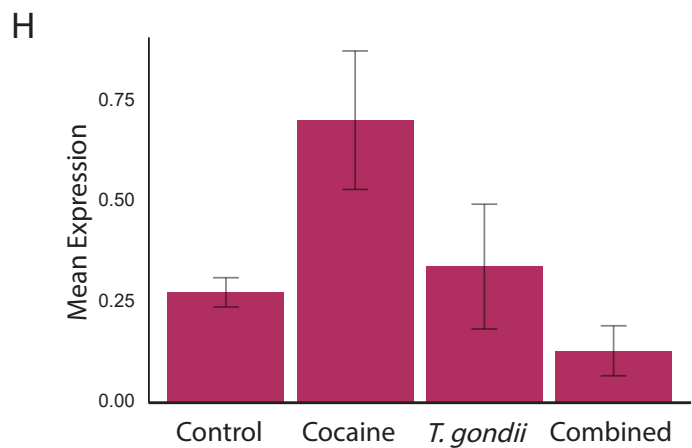

Supplement: S3 Fig — Expression patterns of interacting DE genes by functional effect group (A to H). Significant differences are indicated by an asterisk (Chi square; p < 0.05). (PDF) [file pntd.0010600.s003.pdf]

# hippocampus - 40 genes with significant (FDR <0.1) toxo by cocaine interaction

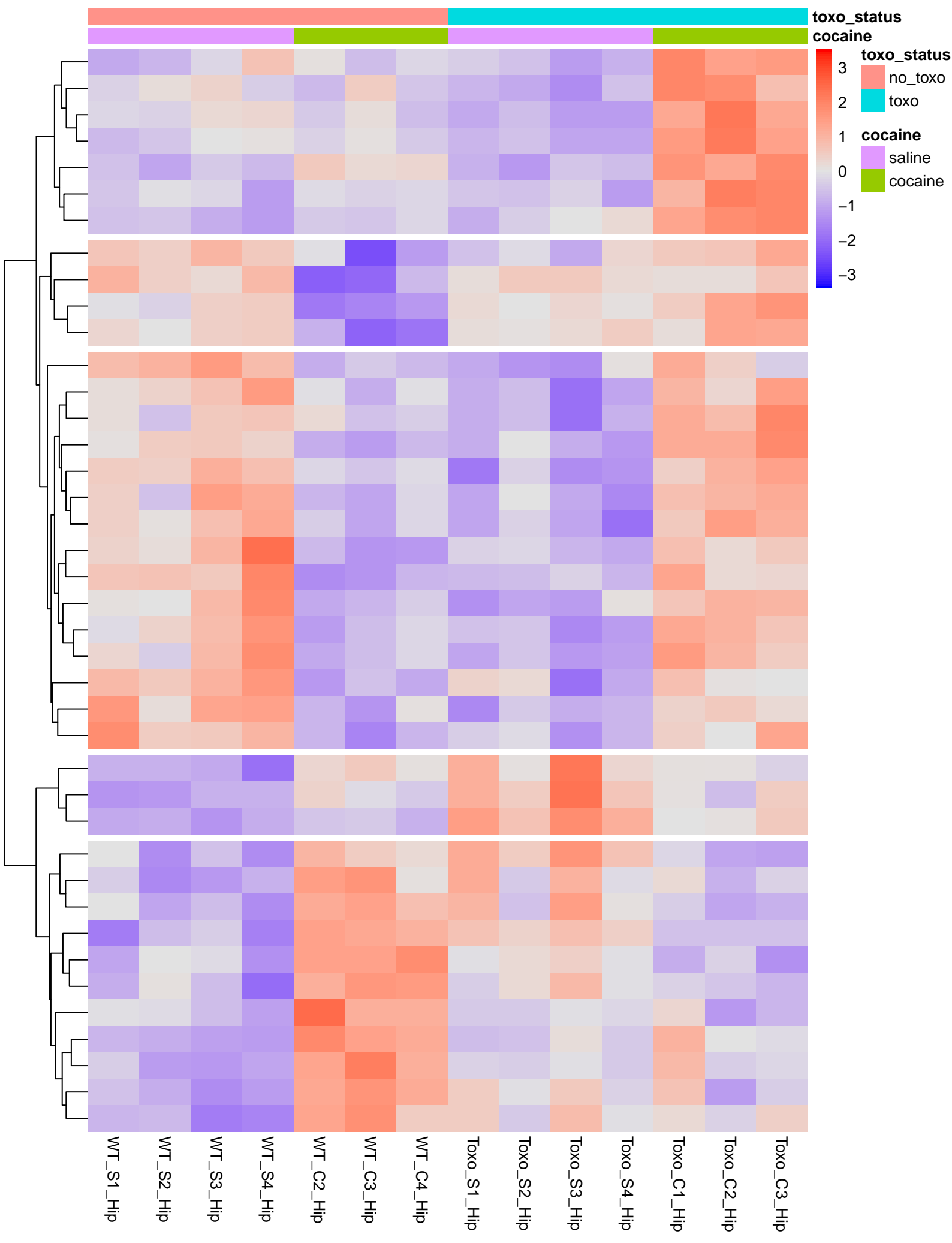

Supplement: S4 Fig — Hippocampus– 40 genes with significant (FDR<0.1) T. gondii by cocaine interactions. (PDF) [file pntd.0010600.s004.pdf]

hippocampus – 364 genes with significant (FDR <0.1) both toxo and cocaine effects

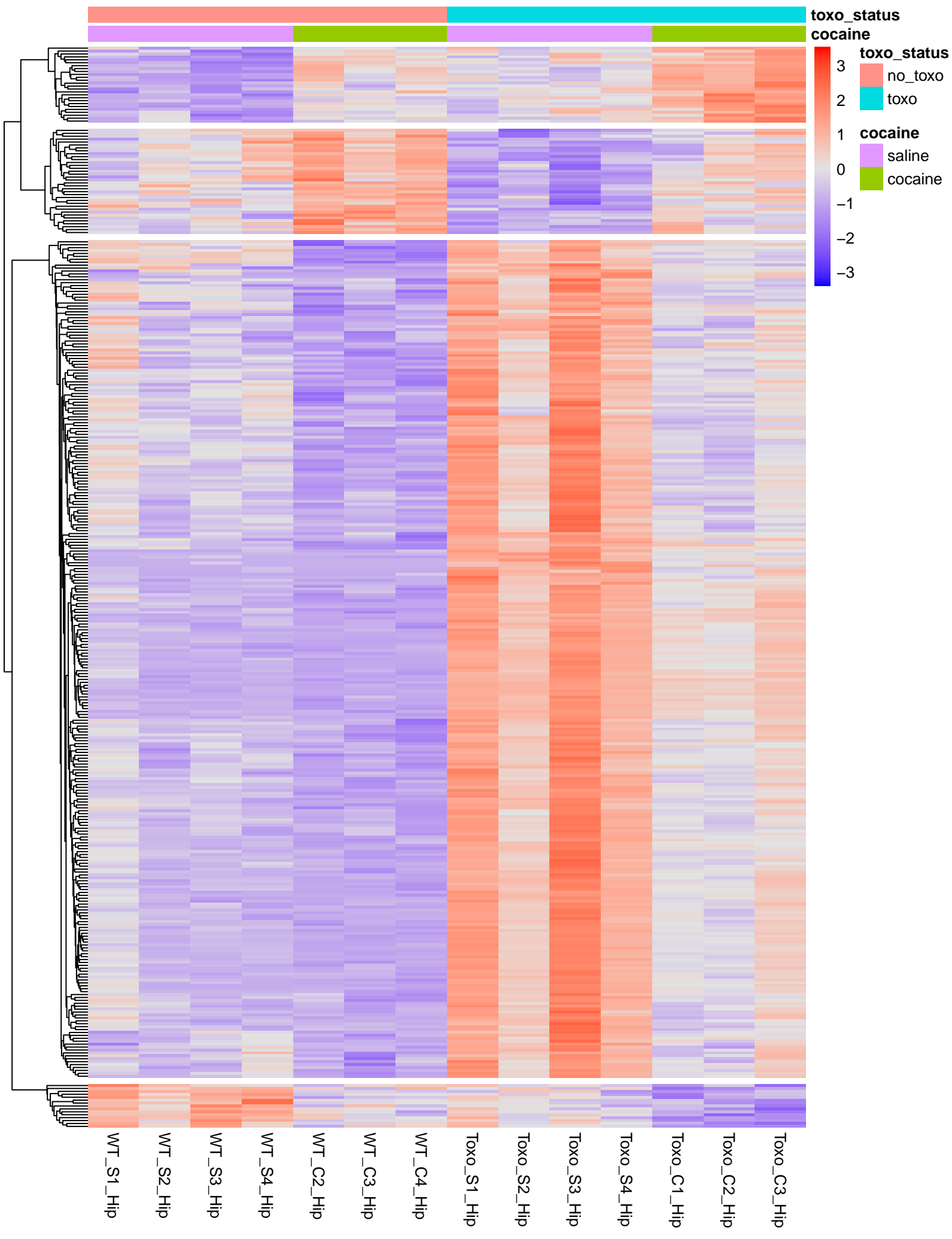

Supplement: S5 Fig — Hippocampus, 364 genes with significant (FDR<0.1) T. gondii and cocaine effects. (PDF) [file pntd.0010600.s005.pdf]

hippocampus - 1143 genes with significant (FDR <0.1) toxo effect only

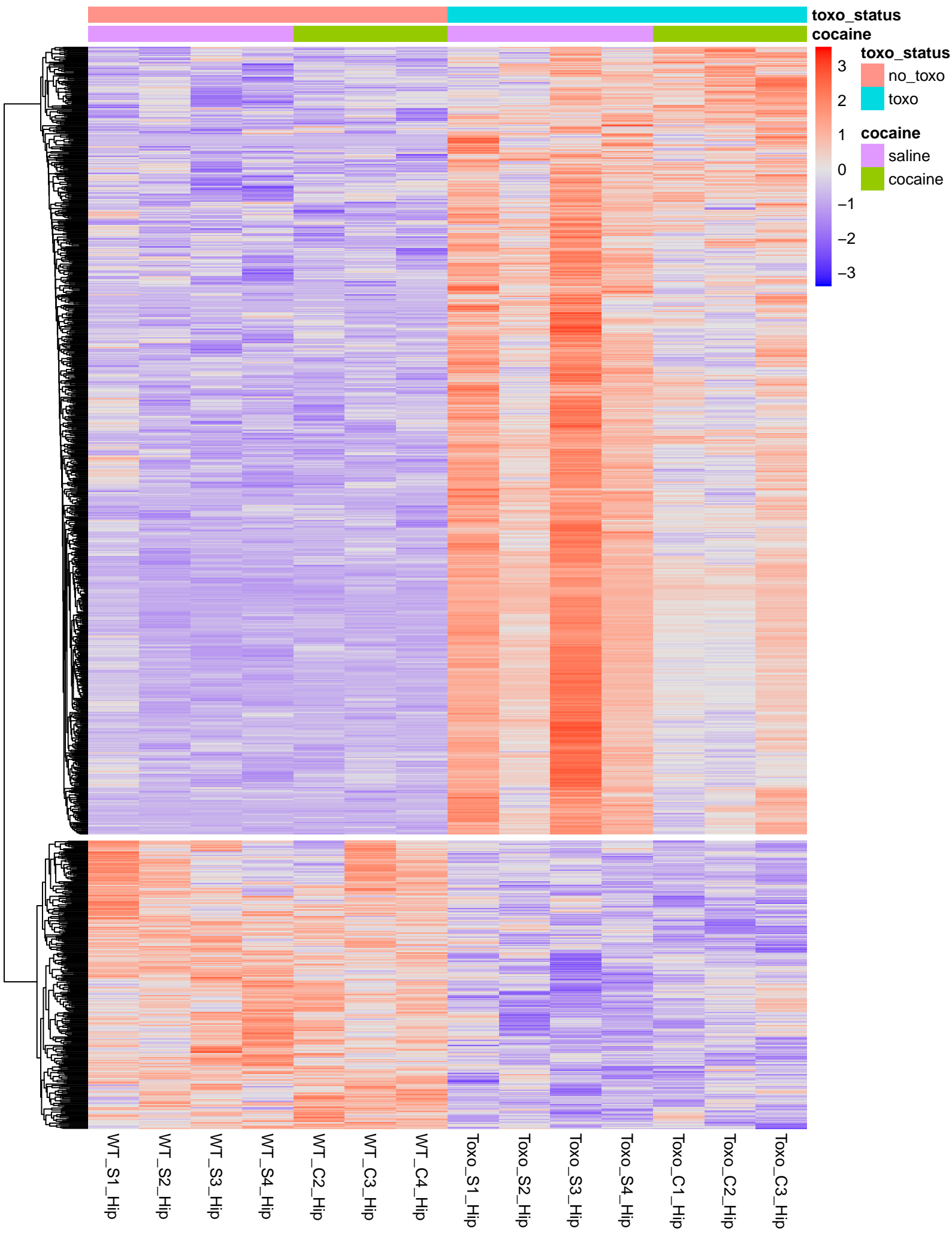

Supplement: S6 Fig — Hippocampus, 1143 genes with significant (FDR<0.1) T. gondii (only) effects. (PDF) [file pntd.0010600.s006.pdf]

hippocampus - 1365 genes with significant (FDR <0.1) cocaine effect only

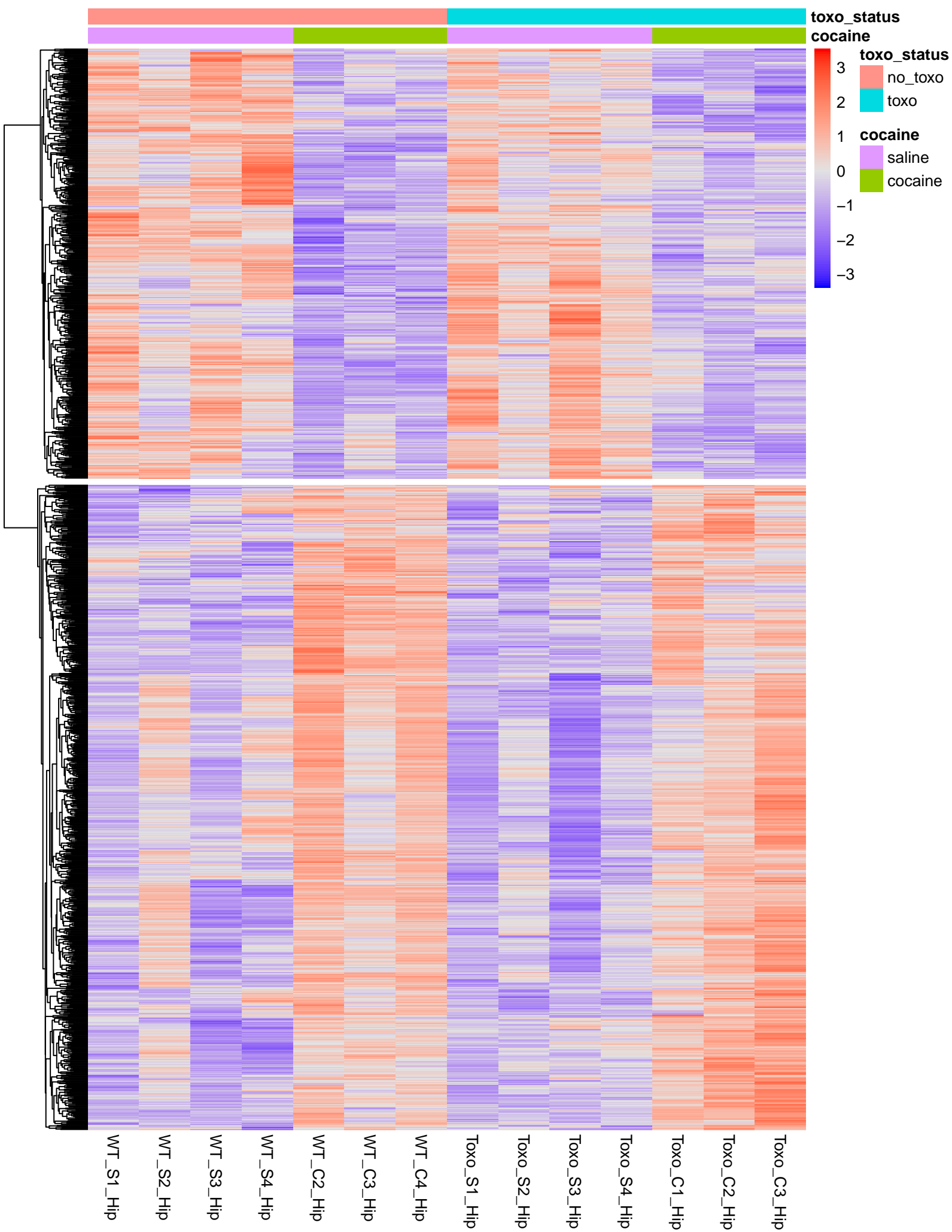

Supplement: S7 Fig — Hippocampus, 1365 genes with significant (FDR<0.1) cocaine (only) effects. (PDF) [file pntd.0010600.s007.pdf]

striatum - 165 genes with significant (FDR <0.1) toxo by cocaine interaction

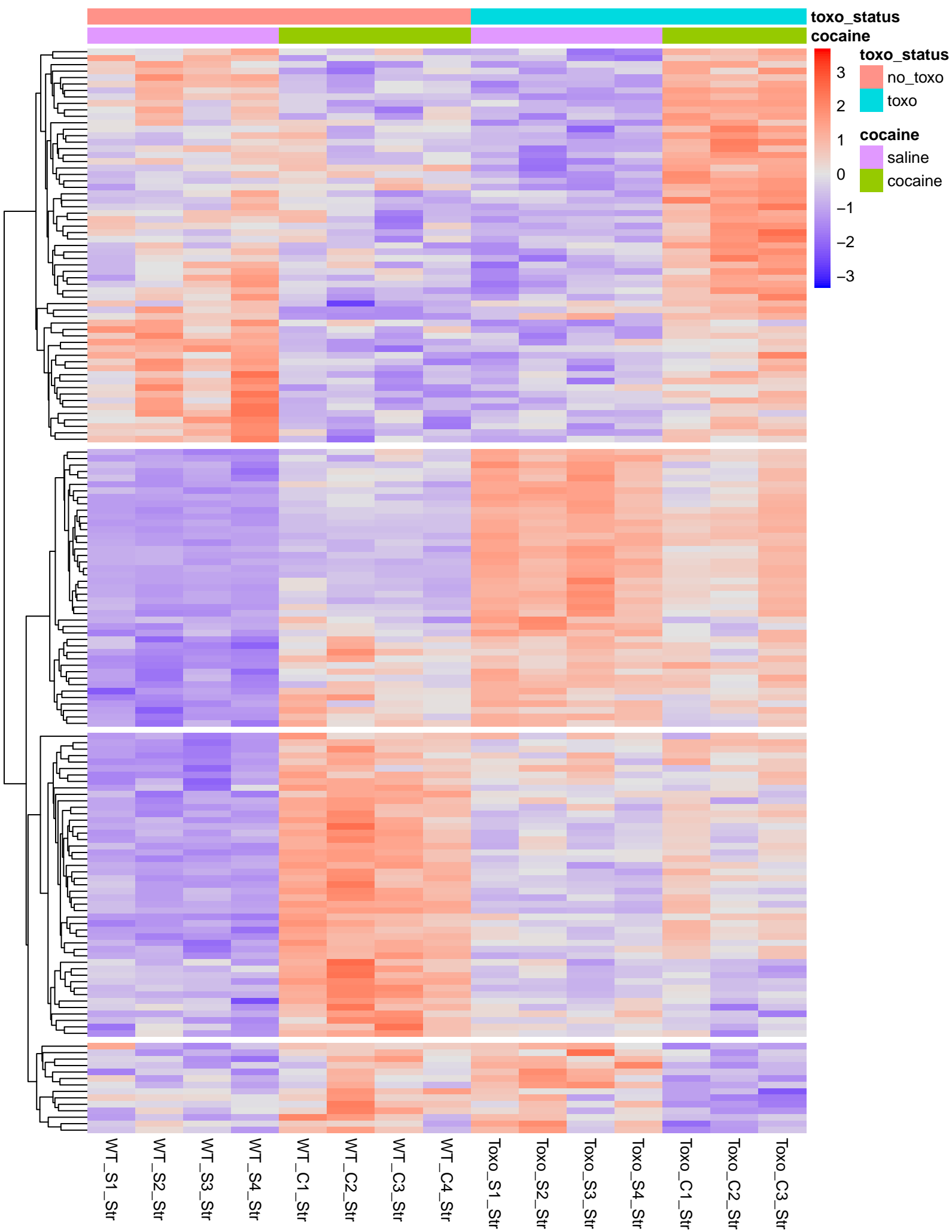

Supplement: S8 Fig — Striatum, 165 genes with significant (FDR<0.1) T. gondii by cocaine interactions. (PDF) [file pntd.0010600.s008.pdf]

striatum - 173 genes with significant (FDR <0.1) both toxo and cocaine effects

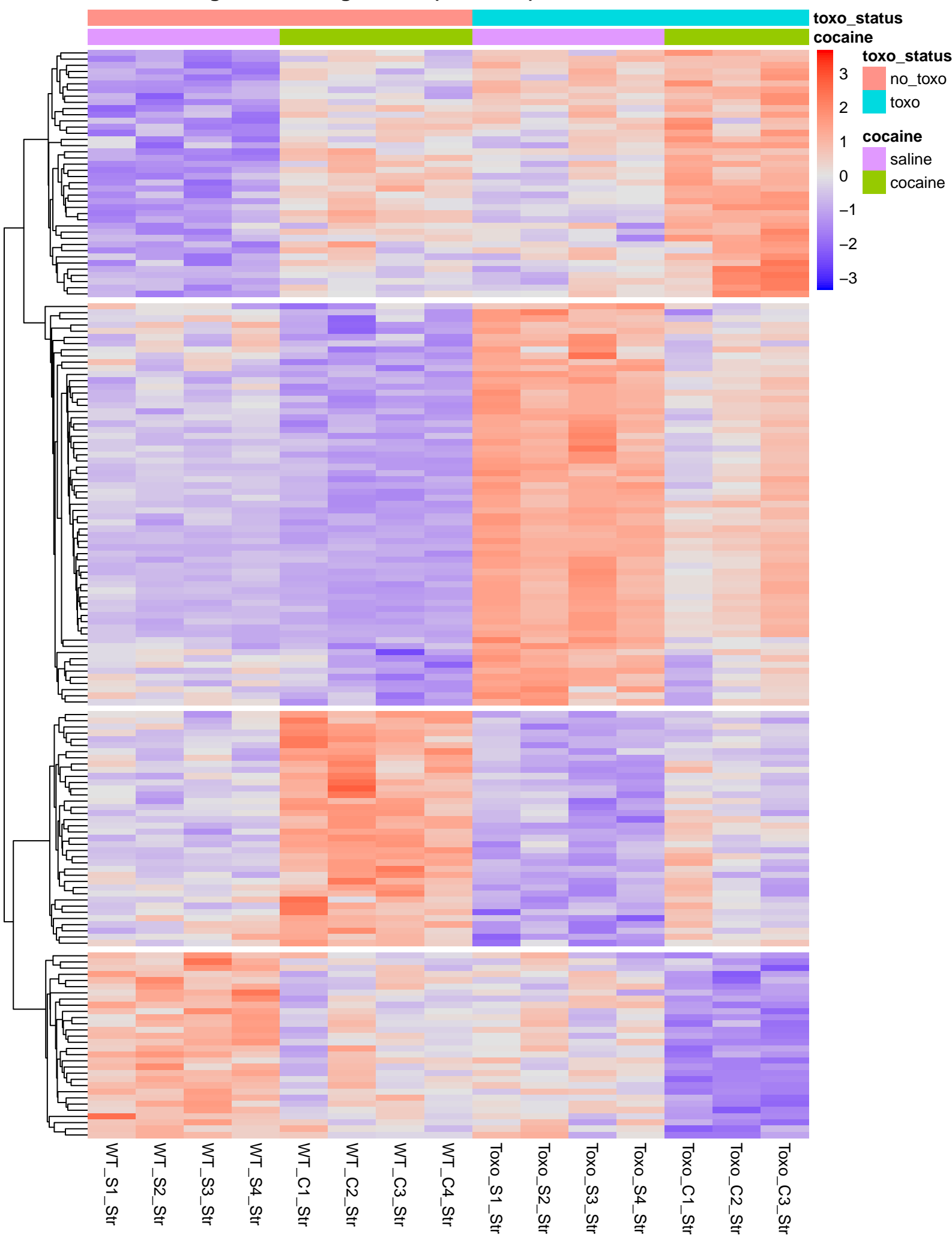

Supplement: S9 Fig — Striatum, 173 genes with significant (FDR<0.1) T. gondii and cocaine effects. (PDF) [file pntd.0010600.s009.pdf]

striatum - 1819 genes with significant (FDR <0.1) toxo effect only

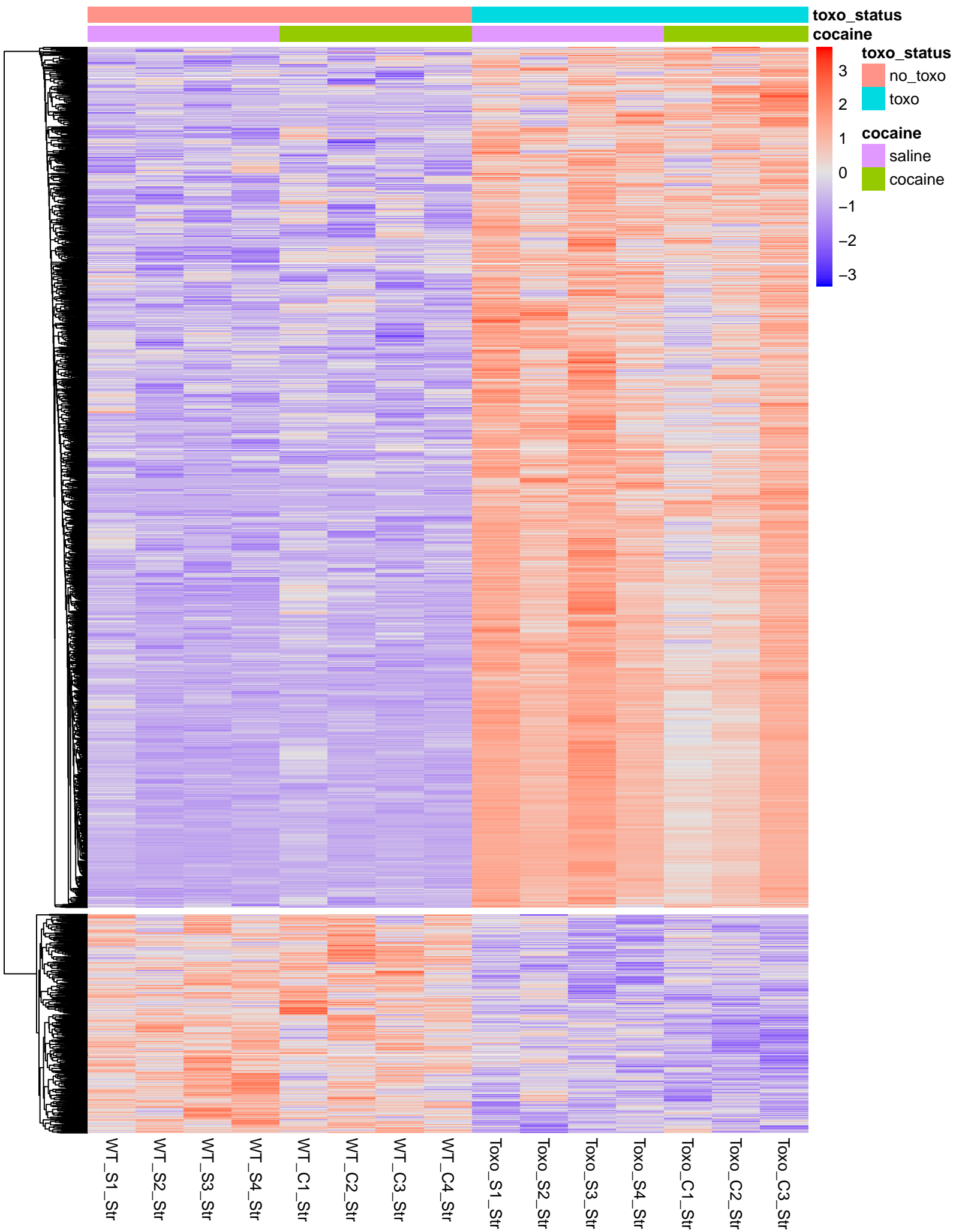

Supplement: S10 Fig — Striatum, 1819 genes with significant (FDR<0.1) T. gondii (only) effects. (PDF) [file pntd.0010600.s010.pdf]

striatum - 920 genes with significant (FDR <0.1) cocaine effect only

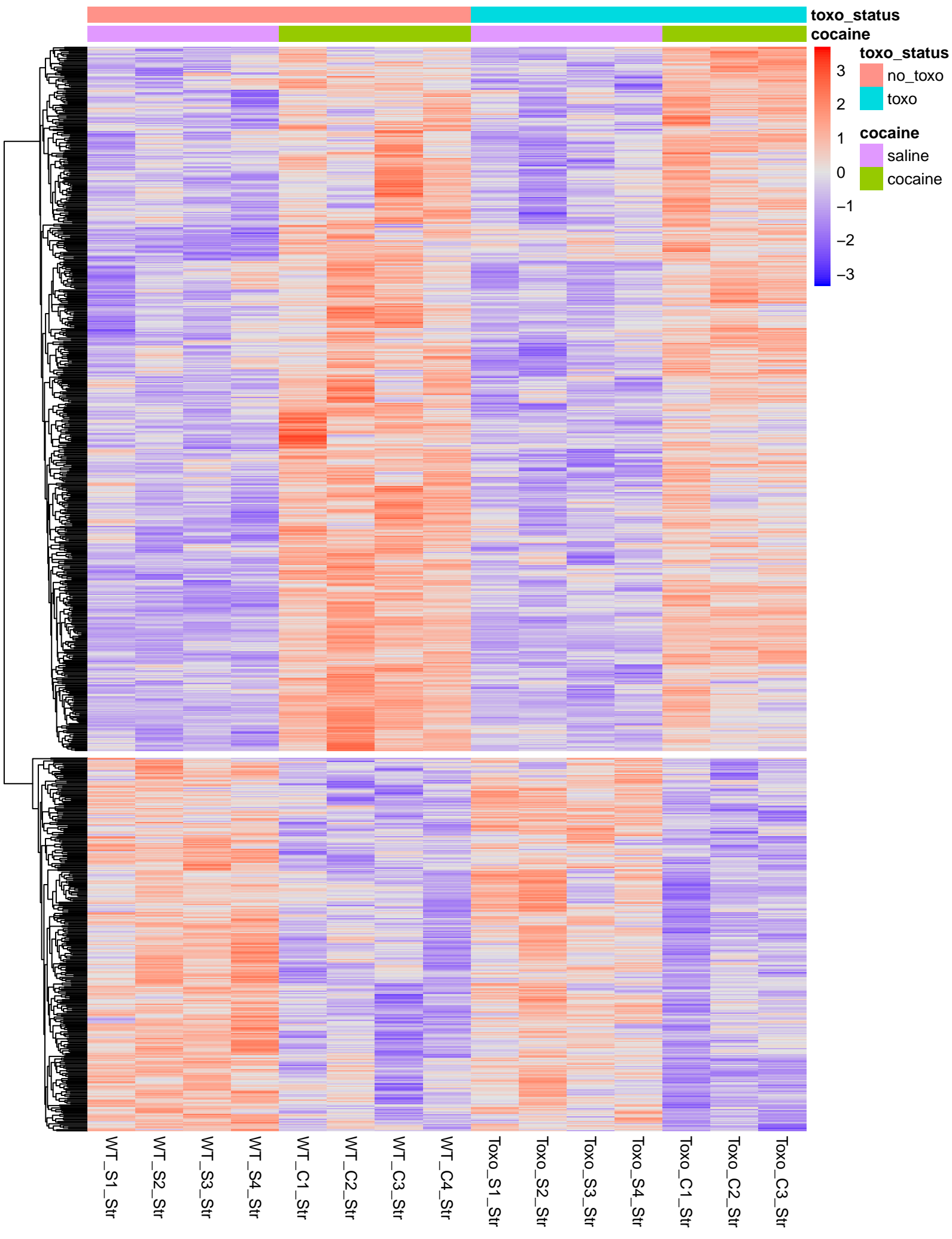

Supplement: S11 Fig — Striatum, 920 genes with significant (FDR<0.1) cocaine (only) effects. (PDF) [file pntd.0010600.s011.pdf]
