# Supplementary material for: Toxoplasma infection in male mice alters dopamine-sensitive behaviors and host gene expression patterns associated with neuropsychiatric disease
Source: PLoS Negl Trop Dis. 2022 Jul 20;16(7):e0010600. doi: 10.1371/journal.pntd.0010600 (PMC9342775; doi:10.1371/journal.pntd.0010600)
Supplement: Attachment — Submitted filename: Response.docx [file pntd.0010600.s022.docx]

June 6^th^, 2022

Professor Bruce A. Rosa,

Associate Editor, *PLoS Neglected Tropical Diseases*

RE: PNTD-D-22-00218

***“Toxoplasma* infection in male mice alters dopamine-sensitive behaviors and host gene expression patterns associated with neuropsychiatric disease**”

Dear Professor Rosa,

We would like to thank you for taking the time to consider our previous submission for publication in *PLoS Neglected Tropical Diseases* and present a revised manuscript in light of your own as well as the reviewers comments. As requested we provide a response that addresses each of the concerns that were previously raised. In addressing these comments we believe that the paper has been significantly strengthened.

In line with journal policy on data requirements, the sequencing data associated with this study have been deposited in the NCBI BioProject database with the BioProject number: PRJNA750929. Additional datasets used in this study have been submitted as supplemental files. Furthermore, we have screened each Figure using the PACE digital diagnostic tool and are happy to discuss further any additional concerns you may have regarding the figures. We look forward to hearing from you and would like to thank you again for taking the time to consider our submission.

Yours Sincerely

Dr. John Parkinson

Senior Scientist - Molecular Medicine, Hospital for Sick Children

Professor - Depts. Biochemistry & Molecular Genetics, University of Toronto

Phone: 1-416-813-5746

FAX: 1-416-813-4931

Email: jparkin@sickkids.ca

Web: http://www.compsysbio.org/lab

EDITORS COMMENTS:

*The reviewers felt that the manuscript was well-written and that results presented are of “paramount importance”, and at this time only minor revisions are necessary.*

*Some important revisions are suggested, including clarifying some results in the figures and some edits to the discussion. Importantly, reviewer 3 considered it a “major reservation” that more comparisons should be made to prior data from the literature, and made some suggestions for interpretation of the data from Figure 2. Reviewer 1 additionally made several helpful suggestions for improving the structure and presentation of the figures. Please considered these suggested revisions.*

We would like to thank you for these comments and have taken the reviewers concerns very seriously. In considering reviewer 3’s suggestion to delete Fig 5A and 6B, we decided that the choice of the word cloud and network are appropriate for these data and that tables or other graphs would not have the same impact. Nevertheless we would be happy to discuss further if you feel that an alternative form of presenting these data is merited.

REVIEWER'S COMMENTS (all comments have been merged for each Reviewer):

Reviewer #1:

***C1:*** *This manuscript details the authors development of a novel mouse model to examine the effects of cocaine treatment on mice with a chronic infection of the parasite Toxoplasma gondii. They perform the series of behavioral tests and find that T. gondii infection blunts the stimulatory effects of cocaine treatment. They then performed RNAseq of the hippocampus and striatum of mice with and without T. gondii chronic infection, and with and without cocaine treatment. Extensive expression and GO analyses are performed on these datasets. While no direct follow-up studies were performed yet, interesting functional effect groups were identified for future studies. Overall the manuscript is extremely well written but the figure and legends are not always clear, so suggestions were made for improvement.*

**A1.** We would like to thank the reviewer for their supportive and highly constructive comments.

***C2*** *- It is not clear in the methods or figure legend what the behavioral sensitization paradigm test is performed for figure 2 panels G and H. It is especially confusing because it is labeled the same as the open field test in panel E but no differences were seen between T. gondii infected and uninfected mice treated with vehicle only. It is unclear why there are no differences in this test in response to T. gondii infection.*

**A2.** We apologise for the confusion and have clarified the methodology for these tests and the interpretation of their results. Behavioral sensitization was measured as the change in locomotor activity in response to cocaine administration. The open field test (former panel E – Now panel F) was performed prior to the cocaine sensitization experiments. In addition, prior to the cocaine treatment the mice were given three days of habituation to the open field. During the first habituation session there was a difference in in distance travelled but this disappeared during sessions 2 and 3. As a result of this habituation there was no further baseline difference in distance travelled in the cocaine sensitization test. This habituation data has been added as Figure 2G. See lines 170-174 & 612-633.

***C3*** *- The dots on figure 3 are very confusing. Are the dots supposed to align with the conditions on the left? The number of affected genes do not match up. For example, is the first dot for Toxo Up? If so why are the number of affected genes in the hippocampus the same (837 vs 1150). Maybe a Venn Diagram would be clearer? Then the reader could easily see how many genes are shared between the various conditions.*

**A3.** We appreciate the concern regarding Figure 3. We chose to use an upset plot over a four-way Venn as we believe it conveys the most meaningful set of data from these analyses. The upset plot combines the features of a histogram (the left-hand bar chart) with a detailed map characterizing overlapping regions corresponding to a traditional multi-way Venn diagram (vertical columns).  The upset plot allows juxtaposition of the same information for both tissues. The bars to the left indicate the total number of differentially expressed genes associated with each of the four conditions. For example, for the bar corresponding to “Toxo Up”, there are 1555 differentially expressed genes in Striatum and 1150 DE genes in Hippocampus that are upregulated in response to Toxoplasma infection, 979 of which are common to both tissues. The filled in dots in the dot matrix, together with the corresponding vertical bars then show how these genes are distributed, thus of the 1150 differentially expressed genes in the Hippocampus associated with “Toxo Up”, 837 are only associated with the “Toxo Up” dataset (1^st^ column), while 287 are shared with the “Cocaine Down” dataset (5^th^ column) and 26 are shared with the “Cocaine Up” dataset (7^th^ column). We have amended the figure legend to clarify the interpretation of this figure. See lines 1126-1135.

***C4:*** *For figure 5, the term descriptions are not that long, so it would be better to have them up in the figure instead of just having the letter code. It is difficult for the reader to go back and forth with the key if want to look at several term descriptions.*

**A4.** As recommended, we have now added the term descriptions to the figure. See Figure 5.

***C5:*** *The term “combined” referring to T. gondii and cocaine together is very confusing in figure 6 as it is not described in later in the text. From the figure legend it reads that combined is hippocampus and striatum. If you want to use the term combined define it clearly in the figure legend and in the results test right away, but it would likely be best to just write Cocaine/T. gondii instead of combined.*

**A5.** As suggested we have replaced combined with “Cocaine+T. gondii”. See Figure 6.

Reviewer #2:

***C6:*** *This manuscript (MS) reports interesting findings that Toxoplasma infection in male mice alters dopamine-sensitive behaviors and host gene expression patterns associated with neuropsychiatric disease. This novel mouse model may present a new perspective to elucidate the molecular pathways by which T. gondii infection contributes to neuropsychiatric disorders.*

*This study was properly designed and the experiments were meticulously performed. The MS is well-written, and the conclusions are supported by the presented data. This MS should be accepted in its present form.*

**A6.** We would like to thank the reviewer for taking the time to read over and lend their support to the study.

Reviewer #3:

***C7:*** *Overall a well thought out and conducted study providing clarity on a complex and interesting topic. A novel approach is applied that could benefit both SZ research and understanding chronic Toxoplasma infection and its link with SZ.*

**A7.** Again we would like to thank the reviewer for their supportive and constructive comments.

***C8:*** *A major reservation that needs addressing in revised versions is comparing with prior data.*

*In Fig 2, the increased time in open zones and increased movement with infection, as found in other studies, is not reproduced. The decreased distance travelled in infected mice may be due to sickness phenotypes and hence wellness and fitness of infected animals should be reported. There is some concern that sickness phenotypes could account for the lack of response for cocaine in Fig 2G. Particularly with the extremely high dose of infection used. Further, could the differences from published behaviour studies be accounted for by Tg strain differences since these are Type III?*

**A8.** We would like to thank the reviewer for raising this important comment. We agree with the reviewer that mouse sickness might introduce a confound in interpreting the open field, and, possibly, the behavioural sensitization and note this in the revised manuscript. While reduced locomotion in the open field is consistent with such an account, we noted no overt signs of ill-health in *T. gondii*-infected mice (e.g., weight loss, lethargy etc). Moreover, normal performance in other behavioural-tests (contextual fear conditioning, elevated plus maze) is generally inconsistent with a sickness based account. To address these points, we now include the distance travelled in the elevated plus maze to figure 2D which, shows no significant difference between groups, as well as a new figure 2K which shows no difference in body weight between groups. Both of these points are generally inconsistent with sickness as a mediating factor and instead suggest that *T. gondii* strain differences might account for differences with previous studies.

We have added a brief discussion to clarify these important points. See lines 161-168; 194-199 & 442-450).

***C9:*** *Lines 91-93 Rewrite sentence because the neurological effects during AIDS may be due to cyst reactivation and active brain cell lysis.*

**A9.** We have revised this sentence in line with the reviewer’s comment to:

“Interestingly, in AIDS patients, immune suppression in the context of T. gondii infection can result in cyst reactivation and active brain cell lysis. Such effects may contribute, at least in part, to psychiatric disturbances such as delusions, auditory hallucinations and thought disorders in as many as 60% of patients [18].” See lines 91-94.

**C10:** line 98-103 Include the NE changes found two studies that impacts cytokine levels in the CNS.

**A10.** We interpreted this comment as a need to provide additional citations regarding the impact of cytokine levels in the CNS, driven by *T. gondii* infection. In line with this comment we have rewritten this section to:

“It is known that chronic infection with T. gondii results in sustained inflammation and disruption of the blood brain barrier, facilitating entry of circulating cytokines into brain tissue [24]. Further, cytokines such as interferon-gamma (IFN-γ), interleukin-6 (IL-6), IL-12b, and tumor necrosis factor (TNF) are considered signatures of T. gondii infection in the CNS of mice [25] and have been positively correlated with cyst load and behavior [8]. In the neuroinflammatory model, this leakage of cytokines into brain tissue results in the recruitment of immune cells leading to long-term neurophysiological changes like those observed in schizophrenia and other disorders such as autism, and bipolar disorder [3, 8, 26].”

See lines 99-106.

***C11:*** *Line 129-138 Delete from Intro as these are Results.*

**A11.** We have removed the text as suggested

***C12:*** *Delete Fig 5A and 6B. Pleasing to the eye but better represented by tables or graphs with error bars and values.*

While we appreciate the comment, we do nonetheless consider that both Fig 5A and 6B do present data in a way that may be more readily interpreted by the reader. Figure 5A, for example, represents a word cloud, an intuitive, visual representation of word frequency. In a word cloud, the more commonly the term appears within the text being analysed, the larger the word appears in the image generated. Word clouds are increasingly being employed as a simple tool to identify the focus of written material. An analogous visual representation are sequence logos, which are widely accepted for visualization of amino acid frequencies in sequence motifs, in preference to histograms. For figure 6B, which represents KEGG pathway term enrichment, we decided to employ a network view to represent the hierarchical structure of the terms, which we believe provides a more intuitive interpretation of our results. For example, a series of parent-child terms with the same enrichments down a branch would be interpreted as being less relevant than a branch where leaf nodes have high enrichment compared to their parent terms. At the same time, in line with the reviewer’s comment, a table of results is available through the Cytoscape file provided in supplemental. In light of the reviewers’ comments, we have added additional text to the figure legends to better clarify the use of these figures. See lines 1142-1146 & 1152-1155.

While we would prefer to keep these figures, we would nevertheless be happy to work with the Editor and Copy Editor if they believe changes are needed for these graphics

**C13:** Line 417-434 This last analysis can be deleted.

**A13.** In revisiting this analysis we did consider it noteworthy that two of these groups of genes are upregulated under exposure to cocaine, but exhibit lower expression in combination with *T. gondii* infection. GSEA revealed several of these genes to be associated with activities that can damage the host hence suggesting that the parasite may be modulating some protective response. Thus rather than delete this analysis, we instead decided to shorten the text. See lines 420-429.
