# Supplementary material for: Toxoplasma infection in male mice alters dopamine-sensitive behaviors and host gene expression patterns associated with neuropsychiatric disease
Source: PLoS Negl Trop Dis. 2022 Jul 20;16(7):e0010600. doi: 10.1371/journal.pntd.0010600 (PMC9342775; doi:10.1371/journal.pntd.0010600)
Supplement: Attachment — Submitted filename: Response_V2.docx [file pntd.0010600.s023.docx]

June 15^th^, 2022

Professor Bruce A. Rosa,

Associate Editor, *PLoS Neglected Tropical Diseases*

RE: PNTD-D-22-00218

***“Toxoplasma* infection in male mice alters dopamine-sensitive behaviors and host gene expression patterns associated with neuropsychiatric disease**”

Dear Professor Rosa,

We would like to thank you again for taking the time to consider our submission. As requested we have made the following changes:

- Improved the alignment of the x-axis labels in Figure 4B such that the last letter aligns to the middle of the two bars
- Removed the letter indicators in Figure 5B as suggested and moved the P-value ranges to the figure caption
- Integrated the supplementary methods into the main text (lines 697-732)

Yours Sincerely

Dr. John Parkinson

Senior Scientist - Molecular Medicine, Hospital for Sick Children

Professor - Depts. Biochemistry & Molecular Genetics, University of Toronto

Phone: 1-416-813-5746

FAX: 1-416-813-4931

Email: jparkin@sickkids.ca

Web: http://www.compsysbio.org/lab
